# Adapting to climate change in South East Queensland, Australia

**DOI:** 10.1007/s10113-013-0505-8

**Published:** 2013-11-28

**Authors:** Ryan R. J. McAllister, Timothy F. Smith, Catherine E. Lovelock, Darryl Low Choy, Andrew J. Ash, Jan McDonald

**Affiliations:** 1 CSIRO Ecosystem Sciences, PO Box 2583, Brisbane, QLD 4001 Australia; 2 Sustainability Research Centre, University of the Sunshine Coast, Maroochydore DC, QLD 4558 Australia; 3 School of Biological Sciences, University of Queensland, St Lucia, QLD 4072 Australia; 4 School of Environment, Griffith University, Nathan, QLD 4111 Australia; 5 Faculty of Law, University of Tasmania, Private Bag 86, Hobart, TAS 7001 Australia

## Introduction

There is growing recognition that regionally scaled responses will be pivotal in adapting to climate change (e.g. Kirshen et al. 2008; Reyer et al. 2012). This recognition is echoed in South East Queensland (SEQ), where rapid population growth and coastal urban centres have made it one of Australia’s most vulnerable regions and a focus for climate adaptation research. As a collection of papers, this special edition contributes to the emerging evidence base for adaptation in the region, but more broadly, the various contributions draw together disciplines and theories that provide lessons for future regionally scaled adaptation studies.

## Why here, why now?

In 2007, discussions began between CSIRO (the Australian Government Research Organisation) and the Queensland Government to better understand climate change vulnerability in SEQ, Australia, and to explore practical and effective adaptation options to manage the risks. These discussions took place in response to a converging set of priorities. At the time, the Queensland Government was looking to implement its Climate Smart Adaptation Strategy, ‘to enhance the State’s resilience to the impacts of climate change’. In parallel, the statutory SEQ Regional Plan was in revision and sought to incorporate climate change into the Regional Plan through a SEQ Climate Change Management Plan.

**Fig. 1:**
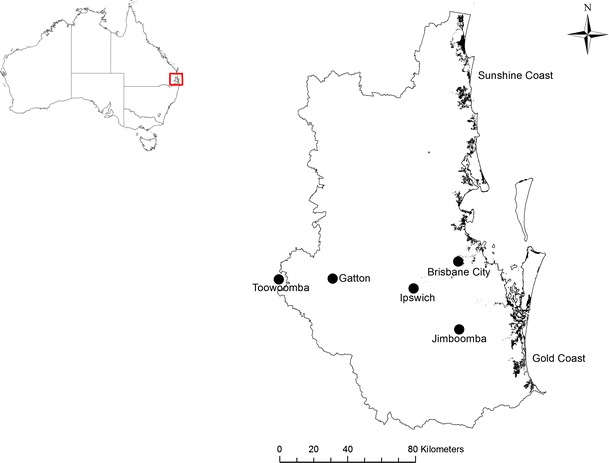
South East Queensland, Australia (coastal shading showing the extent of inundation from a 1.86 m storm surge—a possible 100ARI by 2050, Credit Yong Bing Khoo).

SEQ was an important region in which to develop appropriate adaptation strategies as it was identified as the highest priority region by the Prime Minister’s Science, Engineering and Innovation Council report on Climate Change and Regional Australia (PMSEIC 2007). This high priority ranking considered not only biophysical exposure to climate change, but also the region’s increasing economic and social exposure and sensitivity over the coming decades. By 2007, SEQ was Australia’s fastest growing metropolitan region. In 2011, the population was 3.2 million and this is expected to grow to between 4.2 and 5.1 million people by 2031 (see Roiko et al. 2012). The region is large, diverse and institutionally complex, being governed by eleven local government areas. It covers 22,890 km^2^, has 240 km of coastline, and includes a rich diversity of coastal, rainforest, and sub-tropical woodland ecosystems (Fig. 1). While the population is predominantly urban—which drives the regional economy—agriculture and natural ecosystems are also economically and culturally important.

Climate change will affect different sectors in different ways and the ability to adapt to climate change will also vary considerably across sectors. Hence, there are important trade-offs between the options within sectors and between sectors that need to be understood to confidently guide policy, industry, and community responses to climate change. A comprehensive regional adaptation study in SEQ provided the opportunity to examine both sectoral and cross-sectoral adaptation opportunities and trade-offs.

Two important requirements for undertaking such a regional study were to (a) have multidisciplinary expertise in place, and (b) undertake the research in partnership with policy makers, industry, and communities using participatory research approaches. Multidisciplinary research teams were formed drawing on researchers and research teams from various disciplines across a range of universities within the region and in CSIRO. These research teams had experience in both climate adaptation and participatory research and stakeholder engagement—experience considered essential for undertaking research into adaptive capacity, vulnerability, sectoral adaptation options, and cross-sectoral analysis and synthesis.

## Sectoral options

This special edition focuses on cross-sectoral analysis, integration, and stakeholder engagement, but includes a few more sectoral focused papers that exemplify both the underpinning sectoral analyses and also underpin other, more integrative papers in the edition.

### Peak energy

Peak energy demand can cause unscheduled power shutdowns that present health risks and losses in economic productivity. Quezada et al. (this edition) explore regional peak energy supply adaptations in SEQ in the light of the path dependency inherent in complex urban energy systems. Regional peak energy issues are already driven by cooling demand (Seo et al. 2013). Hence, projected warming in SEQ stands to further stress the energy system (Grozev et al. 2011). Adaptation options include retrofitting houses for increased energy efficiency and using centralised remote control to stage controlled shutdowns of air conditioners and other ‘electricity hungry’ devices during peak electricity demand periods. However, Quezada et al. (this edition) take a socio-technical systems view to show that adaptation has become a contested process. The multiple actors along the energy supply chain hold diverging objectives, capacities, and requirements from adaptation. Moreover, as a backdrop to any adaptation option is an historically entrenched energy system not designed for distributed power generation and electric vehicles, which may form part of SEQ’s future energy distribution system (Quezada et al. this edition).

### Biodiversity conservation

In addition to urban infrastructure, the region holds natural resources that underpin both ecosystem services and the agricultural industry. Shoo et al. (this edition) summarise key regional climate adaptation options for terrestrial and coastal ecosystems, suggesting that strategies for promoting mobility of ecosystems and species, and removing non-climate-related threats, are critical. Identified adaptations include providing room for landward migration of tidal wetlands and increasing spatial connectivity across the full range of bioclimatic variation. The protection of current and predicted refuges from climate extremes is required. Managing the impact of urbanisation on wildlife requires locations of reserves and human settlements to be planned to reduce contact between wildlife and urban pressures, and to accommodate movement of flora and fauna both uphill (to cooler refuges), and landward with sea-level rise (coastal wetlands). To support such adaptations, the authors call for the initiation of long-term studies of species responses to climate change, particularly across ecosystem gradients, and including time-series data on species dynamics (Shoo et al. this edition). Synergies also exist across sectors where less tangible benefits are nevertheless important (Taylor and McAllister this edition). Coastal inundation remains a threat for maintaining existing coastal biodiversity in SEQ. Instilling a preference for soft coastal defences would help protect biodiversity-rich wetlands and maintain amenity as sea levels rise. Upstream buffer zones around wetlands will also allow coastal ecosystems to migrate (Traill et al. 2011).

### Urban water security and flooding

Laves et al. (this edition) examine the challenges of delivering urban water in a fast-growing region where future water supplies will be less secure. This demonstrates one case where research has played a critical role in shaping regional adaptation. While rainfall projections are uncertain, warming and lowering of soil moisture will result in reduced rainfall run-off into water catchments. A portfolio approach to adaptation is already being implemented in SEQ, which is diversifying the region’s dam-based supply system to include treated wastewater for recycling into the potable system, desalination of seawater, and demand management through behavioural change. Today’s decisions for water cycle management establish path dependencies that will shape the effectiveness of adaptation for decades, so special care is needed to avoid maladaptation. Even though more energy is used when using potable water (dishwashers, etc.) than in supplying it, regional adaptations will nevertheless increase the energy intensity of the water system and hence potentially risk increasing greenhouse gas emissions (Laves et al. this edition).

The management of urban water supplies in the region is inextricably linked with management of floods. Bohensky and Leitch (this edition) analyse community perceptions of the links between climate change, flooding, and infrastructure management. One of the region’s worst recorded droughts was broken by major regional flooding events in January 2011. The event took 35 lives in SEQ, shut down the central business district, and flooded around 20,000 houses. Public reaction provided a yardstick of the community’s willingness to adapt in response to natural hazards. Bohensky and Leitch (this edition) systematically analysed how the event was reported in the media, both during the flood event and at its first anniversary. Much of the media and public response cast the flood in terms of blame and political opportunity. The authors suggest that learning was limited, as shown by inadequate attention being paid to longer-term regional issues. This highlights the need for other mechanisms and actors to lead learning processes.

While not covered in this special edition, emergency management (Low Choy et al. 2012) and adaptation for coastal inundation from storm surge (Wang et al. 2010) were also key areas of adaptation research recently conducted in SEQ.

## Cross-sectoral analysis

Many of the innovations required to adapt to climate change will need to come from a cross-sectoral perspective. In this special edition, several papers address various, frequently interlinked aspects of stakeholder engagement, cross-sectoral analysis, and policy integration.

Integrative projects need a process for research teams to be organised for cross-fertilisation of domain thinking. Taylor and McAllister (this edition) present the process used for the three-year regional study in SEQ that formed the basis of this special edition (McAllister et al. 2012). This process included structured reporting of emerging adaptation options and subsequent series of workshops to: share sectoral perspectives, identify cross-sectoral co-benefits and conflicts, and elucidate institutional interdependencies. Taylor and McAllister (this edition) highlight cross-sectoral implications for exemplar problems of (1) wetland migration, coastal infrastructure, and planned retreat; (2) agricultural viability and terrestrial biodiversity; and (3) urban water security and energy demand. Key overarching messages emerge. In order to implement regional policy responses in an integrated way, new, intermediate levels of governance between the local government and individual local households, or businesses, are needed (Taylor and McAllister this edition). In particular, sub-regional coordination of key policies is needed under the SEQ Regional Plan (e.g. the ‘open-space’ and ‘rural land-use strategies’ and biodiversity conservation policies).

Serrao-Neumann et al. (this edition) highlight the importance of applied cross-sectoral policy integration. Exploring coastal areas, they articulate a process used to develop policies, programs, and actions at the local and regional scale. Their collaborative development of cross-sectoral adaptation options with stakeholders followed a learning-by-doing and doing-by-learning approach. Learning-by-doing relates to the development of theoretical knowledge from practice. From the human settlements research perspective, this was pursued by engaging stakeholders from the outset of, and throughout the research. Doing-by-learning relates to the development of practical knowledge from theory. This was conducted in parallel, by reframing adaptation options proposed in the literature in a manner more amenable to adoption and implementation, with reframing guided by stakeholder feedback. The process provides an evidence base to underpinning requirements for adaptation: (1) an informed and confident political, private sector, and community leadership; supported by (2) an informed, engaged, and prepared community; that are (3) reinforced through continuous awareness, training, education and capacity building programs; and that operate in (4) a process of full stakeholder engagement leading to mutually agreed actions.

Finally, in the context of running a regional research agenda with a strong focus on engagement, McAllister et al. (this edition) sought to examine the bigger picture of stakeholder participation in climate adaptation in SEQ. Similar to many other regions, in SEQ, decisions are made and contested across a range of actors with divergent interests (e.g. Taylor et al. in press), where organisations generally act based on their own narrow, strategic interests. McAllister et al. (this edition) tracked organisational participation in policy forums between 2008 and 2012. These forums included research-led forums, as well as government- and NGO-led forums. They used network theory and interviews to understand the nature of stakeholder engagement. They found that stakeholders generally engaged in order to promote their organisation’s interests rather than using these policy networks to source information for decision making in the public interest. While the regional research agenda sought to inform policy, the research relating to the engagement process suggests that cooperation on policy cannot be assumed and that to expand the scope of policy options requires explicit efforts to counterbalance special interest advocacy.

## Feasibility and adaptive capacity options

Keys et al. (this edition) explore adaptive capacity—the factors that influence the effectiveness of SEQ adaptation strategies across particular sectors. They combined four methods of assessment: (1) an assessment of the socio-economic trends affecting the region (Roiko et al. 2012); (2) an historical analysis of adaptive capacity across a range of sectors and scales, including 33 international case studies (Bussey et al. 2012); (3) a series of system conceptualisation workshops across various sectors in SEQ (seven workshops with 66 participants); and (4) interviews with 42 SEQ sector representatives to develop Bayesian belief networks to determine the probability of successful adaptation under various conditions (Richards et al. 2013). The consolidation of this research draws recommendations for building SEQ’s adaptive capacity (Keys et al. this edition). Simultaneous initiatives are required that build: community resilience and well-being; and new institutional and finance capacities. For example, increasing the capacity of socially disadvantaged groups requires simultaneous initiatives that: build networks and social capital, change policies that facilitate shared housing arrangements, and provide financial incentives for implementing adaptive responses. In addition, the intent of adaptation requires critique to avoid inferior path dependencies (Thomsen et al. 2012). Adaptive capacity options also need to be considered across various temporal scales and aligned to current norms, to judge their likely success or failure, and to identify additional, complementary initiatives. In summary, while resources will be critical for adaptation, the primary adaptive capacity challenges for SEQ are likely to be cultural rather than structural.

## Conclusions

The motivation for this special edition was a recent, three-year research project exploring regional climate adaptation in SEQ, Australia. The papers illustrate how the project targeted on-the-ground impact in four key ways (McAllister et al. 2012). First, by direct engagement in local policy networks, incremental changes to how climate change is planned have been supported. Researchers gave more than 70 stakeholder presentations over the course of the project. Second, scientific information has been directly fed into existing policy frameworks. Researchers contributed to six formal policy responses over the course of the project and sat on eight climate change expert panels. Third, political debates have been assisted by the provision of an evidence base on the value of certain adaptation options. Examples include cost-benefit analyses of changing planning codes for inundation risk and building codes for wind (Stewart and Wang 2011; Wang et al. 2010). Fourth, and importantly, a substantial contribution has been made to the climate change literacy of scholars and stakeholders, which builds our collective adaptive capacity to meet future challenges. Eleven climate adaptation focused research positions were created throughout the project, and seven PhD projects were directly supported.

After the project’s inception in 2007, Australia’s so-called Millennium drought broke with devastating SEQ floods in 2011. The project was completed in May 2012, and soon afterwards in 2013, the nation recorded its hottest ever summer while the region experienced more flooding and damaging winds. Our adaptation knowledge base has grown, particularly with regard to the need to consider infrastructure, knowledge, and policy change adaptations in unison. Meanwhile, government, policy, and economic conditions have all shifted in recent years in ways that have slowed the push to adapt for regional climate change in SEQ. While the future will always be uncertain, the need for adaptation is compelling. With local context being a key driver in deciding which adaptations are most appropriate, the regional scale will continue to be a key focus of attention.
